# Recent Advances in RAFT Polymerization: Novel Initiation Mechanisms and Optoelectronic Applications

**DOI:** 10.3390/polym10030318

**Published:** 2018-03-14

**Authors:** Xiangyu Tian, Junjie Ding, Bin Zhang, Feng Qiu, Xiaodong Zhuang, Yu Chen

**Affiliations:** 1 Key Laboratory for Advanced Materials and Shanghai Key Laboratory of Functional Materials Chemistry, Institute of Applied Chemistry, East China University of Science and Technology, 130 Meilong Road, Shanghai 200237, China; y20150070@mail.ecust.edu.cn (X.T.); y20160051@mail.ecust.edu.cn (J.D.); zhangbin@ecust.edu.cn (B.Z.); 2 The State Key Laboratory of Metal Matrix Composites & Shanghai Key Laboratory of Electrical Insulation and Thermal Ageing, School of Chemistry and Chemical Engineering, Shanghai Jiao Tong University, Dongchuan Road 800, Shanghai 200240, China; fengqiu@sit.edu.cn; 3 Center for Advancing Electronics Dresden (CFAED) & Department of Chemistry and Food Chemistry, Technische Universität Dresden, 01062 Dresden, Germany

**Keywords:** RAFT polymerization, chain transfer agent, initiation method, optoelectronic applications

## Abstract

Reversible addition-fragmentation chain transfer (RAFT) is considered to be one of most famous reversible deactivation radical polymerization protocols. Benefiting from its living or controlled polymerization process, complex polymeric architectures with controlled molecular weight, low dispersity, as well as various functionality have been constructed, which could be applied in wide fields, including materials, biology, and electrology. Under the continuous research improvement, main achievements have focused on the development of new RAFT techniques, containing fancy initiation methods (e.g., photo, metal, enzyme, redox and acid), sulfur-free RAFT system and their applications in many fields. This review summarizes the current advances in major bright spot of novel RAFT techniques as well as their potential applications in the optoelectronic field, especially in the past a few years.

## 1. Introduction

Macromolecules with high molecular weight have many advantages in chemical and physical properties over traditional small molecular materials. A general method for preparation of macromolecules is to convert small molecules into high molecular weight polymers by simple organic reactions, such as coupling reaction polymerization [1,2], condensation polymerization [3,4,5], and radical polymerization [6]. Among them, radical polymerization is an internationally recognized polymerization technique, which plays an important role in practical researches and applications [7]. Wide reports have demonstrated a large number of advantages in radical polymerization, like compatibility with a wide range of (meth)acrylates, vinyl acetate, acrylonitrile, styrenes, and *N*-vinylpyrrolidone monomers, facile reaction conditions in bulk, solution and emulsion, and tolerance of many functionalities, including electron-donating groups and electro-drawing [8,9,10,11]. However, a free radical polymerization is repeatable, but thus not allowing microstructural control of high livingness and chain length. Thus, the development of new polymerization technology for controlled molecular weight with complex architecture and novel functionalities is highly desirable.

In 1956, Szwarc firstly reported the living polymerization and described the controlled-polymerization of diene and vinyl monomers [12]. This discovery promotes the development of controlled cationic [13,14], anionic [15,16], as well as ring-opening polymerization [17]. Unfortunately, these polymerization methods often required harsh polymerization conditions, e.g., anhydrous and anaerobic environment [18,19]. Close to radical polymerization, reversible deactivation radical polymerizations (RDRP), such as nitroxide-mediated polymerization (NMP) [20,21], metal-mediated living radical polymerization (LRP) [6], atom transfer radical polymerization (ATRP) [22], and reversible addition-fragmentation chain transfer (RAFT) polymerization [23,24], have drawn much attention due to their facile and simple operating mode. Especially, RAFT polymerization reported firstly by CSIRO in 1998 [23] has now emerged as the most widely used polymerization technique. Due to its living and controllable in the whole polymerization process, RAFT polymerization has been considered as a precious pearl in the fabrication of multiblock copolymer and complex polymeric architectures. The development of RAFT polymerization has gone through two main stages: the proposition and illumination of the mechanism; then, the fabrication of various complex polymeric architectures and functional polymeric materials through the RAFT process for practical applications in different fields. To date, a large number of new RAFT polymerization systems have been reported to improve the polymerization performance of conventional RAFT technique, receiving much attention to scientists for the construction of functional polymers in wide applications.

To date, over hundreds of reviews have been published involving in RAFT polymerization, covering a tremendous amount of polymer synthesis and applications, in terms of architectures (block, star, hyperbranched, cross-linked) [25,26,27,28,29], surface-grafted (inorganic nanocomposites, 2D graphene) [30,31,32] and functionalities (stimuli-responsive, biodegradable) [33,34,35,36]. In these studies, optoelectronic functional materials by RAFT polymerization have been widely investigated for their wide application prospect. The advantages of RAFT polymerization permit the accurate control of optoelectronic polymers in molecular weight, molecular weight distributions and architecture. It will bring great benefits for the improvement of optoelectronic devices performance. However, few reviews of RAFT polymerization summed up its related application in the field of optoelectronics. This review mainly consists of two parts: the first part particularly highlights the latest research of novel RAFT technology, including various initiation methods, e.g., photo, metal, enzyme, redox and acid, and sulfur-free RAFT systems. The optical and electronic applications of functional polymers by RAFT technology are also summarized in second part.

## 2. General Mechanism of RAFT Process

**Scheme 1:**
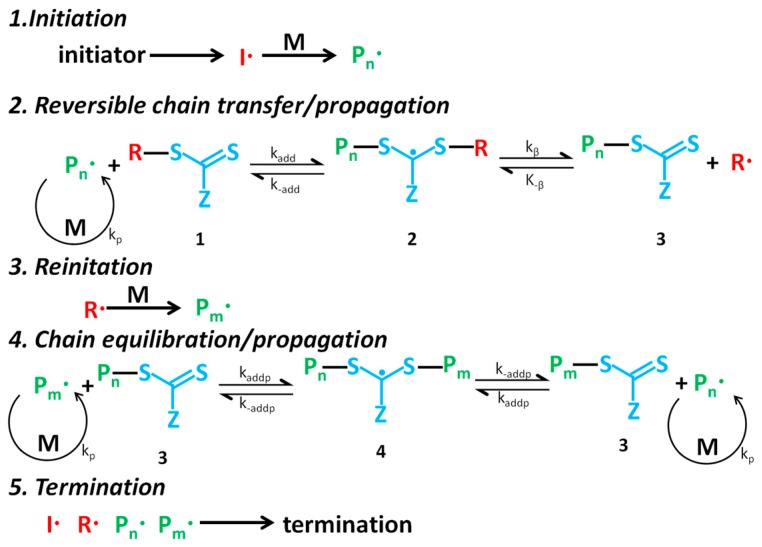
Mechanism of reversible addition-fragmentation chain transfer (RAFT) polymerization.

The currently approved mechanism of the RAFT process is depicted in Scheme 1. Initiation, chain propagation and chain termination in RAFT polymerization are somewhat alike to those in conventional radical polymerization. However, the most basic and important step for RAFT process is reversible addition-fragmentation equilibrium (step 2 and step 4). In a typical RAFT process, radical (**I^•^**) from initiator reacts with monomers (**M**) to give a propagating radical (**P_n_^•^**) (step 1). Subsequently, the propagating radical (**P_n_^•^**) adds to the C=S group originated from RAFT agent (**1**) (chain transfer agent, CTA) and an intermediate radical (**2**) will be generated. Interestingly, the appearance of intermediate radical (**2**) will restrict the irreversible termination reaction between the propagating, and intermediate radical will also give two types of fragmentation benefited from the activity of C=S group. It is possible to return to their initial state to get a propagating radical (**P_n_^•^**) and RAFT agent, while the intermediate radical (**2**) may fragment into a new radical (**R^•^**) and a dormant chain (polymeric thiocarbonylthio compound) (**3**). Then, the new propagating radical (**P_m_^•^**) will be formed after the reinitation monomer polymerization by the new formative radical (**R^•^**) (step 3). In this case, a rapid equilibrium will be formed between the dormant species (**3**) and active propagating radicals (**P_n_^•^** and **P_m_^•^**) (step 2 and step 4), which can enable all of the chains to have equal chance to grow, leading to a similar degree of polymerization. The termination reaction often is inevitable in a radical polymerization system (step 5). For RAFT polymerization, the termination reaction could be minimized, due to the existence of thiocarbonylthio end-group in resulting polymeric chains, which also enables participating in RAFT polymerization as RAFT agent. Using as macroinitiator, other monomers can be polymerized to fabricate multiblock polymers.

**Scheme 2:**
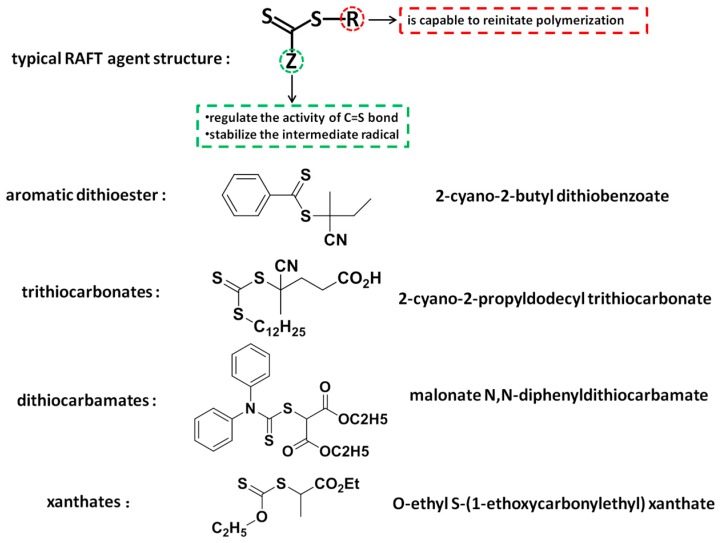
Typical RAFT agent structures and different types of RAFT agents.

As described above, the activity of C=S group in RAFT agent plays a pivotal role in controlling and regulating the polymerization process. Here, the typical types of RAFT agent structures are shown in Scheme 2. The realization of function depends greatly on the substitution of Z- and R-group, respectively. The former Z group often regulates the activity of C=S bond and stabilizes the intermediate radical. Owing to the versatility of monomer compatibility of RAFT, different kinds of Z-groups could be selected to polymerize monomers with different reactivity. At the same time, the latter R-group should be an excellent leaving group when the C=S bond of RAFT agent undergoes the addition of propagating radical, and it also can reinitiate polymerization rapidly and efficiently. In other words, the leaving R-group should have same function and efficiency with the initiator and monomer radical. In brief, an appropriate RAFT agent is vital to achieve controlled and living process in ideal RAFT polymerization. Until now, aromatic dithioester, trithiocarbonates, dithiocarbamates and xanthates are four major categories of typical RAFT agents. Aromatic dithioester and trithiocarbonates are often used to polymerize “More activated” monomers (MAMs), including butadiene, isoprene, styrene, vinylpyridine, (meth)acrylates, (meth)acrylamides, maleic anhydride, maleimide, and acrylonitrile. Z-group of aromatic dithioester and trithiocarbonates can stable the intermediate radical when MAMs radical adds to the C=S bond, attributing to the considerably stable radical generated from MAMs. By contrast, the high reactivity of “Less activated” monomers (LAMs) may produce a relatively stable intermediate radical. Xanthates and dithiocarbamates can deactivate the C=S bond through the delocalization of lone pair of electrons from oxygen in xanthates or nitrogen in dithiocarbamates to thiocarbonyl group, resulting in the destabilization of the radical intermediate for accelerated polymerization. More detailed tutorials for selection of the R-group and Z-group of RAFT agent for various polymerizations could be founded in other reviews [37,38].

The radical is another crucial factor in the RAFT process. The number and concentration of initiators as the parent of radical are closely related to the amount of dead chain and polymerization rate. Kinetic researches demonstrate the maximum number of living chains and the high polymerization rate would be achieved in a low concentration of initiators [39,40,41]. Meantime, monomers with a high propagating rate coefficient and initiators with high decomposition are also achieved. Ordinarily, thermal initiator is preferentially used for RAFT polymerization system, such as azodiisobutyronitrile (AIBN) [42,43,44], 2,2′-azobis-(2,4-dimethylvaleronitrile) (ABVN) [45], or benzoyl peroxide (BPO) [46].

## 3. Novel RAFT Techniques

In general, a conventional RAFT process employs thermal initiator as a radical source and thiocarbonylthio compound as a RAFT agent. The disadvantages of this thermal decomposition initiation system, like side reaction, self-initiation polymerization of monomer, and irreversible chain transfer, greatly limit their wide polymerizations in some special condition. Recently, new strategies of radical generation induced by other means (e.g., photo, metal, enzyme, redox and acid) and some special RAFT process have been reported to optimize the conventional RAFT polymerization.

### 3.1. Photo-Induced Initiation

**Figure 1:**
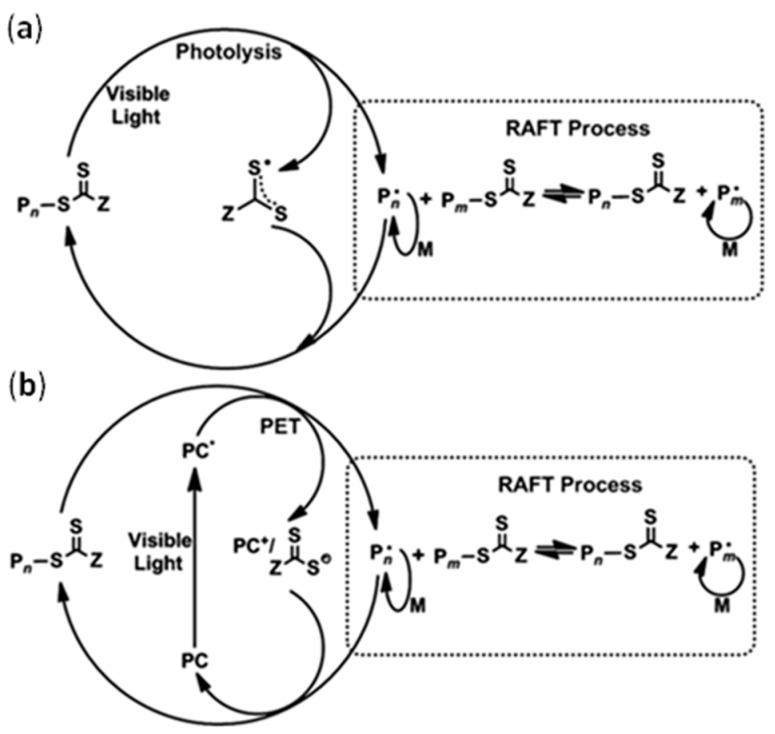
Proposed mechanism for photo-induced initiation RAFT. (**a**) Photodissociation RAFT process; (**b**) photoinduced electron (PET)-RAFT process. Note: PC, photoredox catalyst. Reprinted with permission from [47]. Copyright 2015 American Chemical Society.

In comparison with that of thermal initiation, a simplified RAFT operating process could be developed by photo-induced initiation, in which the polymerization condition is usually at room temperature without the degassing procedure. More importantly, the polymerization process can be facilely controlled through switching the “on” and “off” of light. This unique photo-controlled RAFT polymerization could be grouped into two types: (i) the homolysis of RAFT agent activated directly by light to give the initiated radical **R^•^** instead of the conventional radical from added initiator; and, (ii) the generation of radical initiated by photoinitiator or photoredox catalyst through the electron transfer process under the light irradiation, known as photoinduced electron transfer-reversible addition-fragmentation chain transfer (PET-RAFT) (Figure 1).

Without the addition initiator or catalyst, the direct homolysis of RAFT agent using light can avoid contamination that is caused by the addition of external radical resource, especially for metal complex. In this process, RAFT agent plays the dual roles in both a photoinitiator and a chain transfer agent, called photoiniferter, which can generate the stable tertiary carbon-centered radical under the light irradiation. As shown in Table 1, Xanthates [48], dithioesters [47,49] and trithiocarbonates [50], as photoiniferter have been presented for the polymerization of MMA, MA, and acrylic acid in the early days. A 4-cyano-4-((dodecylsulfanylthiocarbonyl)sulfanyl)pentanoic acid (CDTPA)-derived poly(oligo(ethylene glycol) methyl ether methacrylate) (POEGMA) macro-CTA can also be photodissociated directly to conduct RAFT polymerization of BzMA without any photoinitiator, and the morphologies of polymeric nanoparticles could be regulated frequently by the change of the wavelength of light [51]. The higher polymerization efficiency could be obtained by photoinitiator process than that of the traditional RAFT process. For example, the decomposition rate of photoiniferter in the presence of UV irradiation is faster than that of thermal dissociation rate of AIBN at 60 °C, and the polymerization rate would be increased with the increasing of ultraviolet light intensity [52]. Nevertheless, the high energy of the UV light may lead to an irreversible RAFT agent decomposition and unexpected side reaction [53,54]. Recently, Green light radiation had been confirmed as the safe light source, which has been conducted to control the miniemulsion polymerization of butyl methacrylate successfully [55]. Boyer et al. described a RAFT strategy for the precise synthesis of a well-defined oligomer with five different vinyl monomer through the difference of relative activity when the individual unit monomer inserted in RAFT agent under green light initiation. It implements accurate controllability at the monomer level [56]. However, these polymers bearing a thiocarbonylthio end-group at the ω-end are still sensitive to light and lose the end-group fidelity [53,54].

For PET-RAFT approach, Boyer’s group reported the first RAFT polymerization that was activated by photoinduced electron transfer process coupled with photoredox catalyst [58]. Based on the proposed mechanism, the generation of radical **R^•^** is accompanied by a redox process. Under light irradiation, the RAFT agent is reduced to provide a radical **R^•^**, along with the formation of oxidized photoredox catalyst. This radical **R^•^** initiates monomer to become a propagating radical, and enter the equilibrium of activation/deactivation chain transfer process. Different from the photodissociation RAFT agent, this PET-RAFT system can minimize the formation of dead chain to restrict the irreversible light degradation reaction by not using the exogenous radical initiators [58,59]. To date, thiocarbonylthio compounds with higher redox potential are perfect RAFT agent for reduction and initiation [58], and plenty of photoredox catalysts, such as fac-[Ir(ppy)_3_] [58,60], Ru(bpy)_3_Cl_2_ [61,62], zinc porphyrins (ZnTPP) [63,64], tertiary amine (NR_3_) [65], and Esosin Y [66,67], have been successfully applied to the PET-RAFT polymerization. As a special exception, ZnTPP can activate CPADB (−0.4 V versus SCE) more easily than that of BTPA (−0.6 V versus SCE), may result from the specific coordination activation of trithiocarbonate with ZnTPP [63]. Titanium dioxide (TiO_2_) has also been demonstrated to be a suitable photoredox catalyst to conduct PET-RAFT polymerization of MMA. However, the high energy of UV light would also decompose dithioester after irradiation time for 500 min, leading to the uncontrollable polymerization process [68]. Thus, visible light induced PET-RAFT polymerization systems have attracted more and more attention for researchers [57,69]. The Boyer’s group reported the PET-RAFT polymerization of photosensitive *o*-nitrobenzyl methacrylate without photolysis by-product of monomer/polymer by using ZnTPP or PheoA as photoredox catalyst, respectively, under visible red or yellow light [70]. Recently, the lower reduction potential of Bacteriochlorophyll a showed a large molar absorbability in the near-infrared and far-red region, which could reduce RAFT agent efficiently with low absorption overlap. Using bacteriochlorophyll a as a photoredox catalysts, the polymerizations of MMA and tert-butyl methacrylate with controlled molecular weight (PDI < 1.25) were reported with near-infrared and far-red irradiation. These light resources with excellent penetration would expand the potential application of PET-RAFT polymerization in biomedicine and industry [71]. On the other hand, the high potential for oxygen tolerance in PET-RAFT technology enables it to conduct the polymerization process under the air environment without the degassing procedure by freeze-pump-thaw cycles [63,65,66]. Qiao’s group reported the PET-RAFT polymerization of MA by using *g*-C_3_N_4_/amine as cocatalyst in the air condition, in which *g*-C_3_N_4_ cannot only advance the polymerization rate, but also accelerate the consumption of oxygen through the electron transfer from the amine to dissolved oxygen, which is mainly caused by more negative conduction band of *g*-C_3_N_4_. Interestingly, without the external deoxidant, the thioether group from (methylthio) ethyl methacrylate (MTEMA) monomer can quench photosensitized singlet oxygen in PET-RAFT process, which give no distinction product between the presence and the absence of air [72]. The details for the PET-RAFT are listed in Table 2.

### 3.2. Metal-Catalytic Initiation

**Figure 2:**
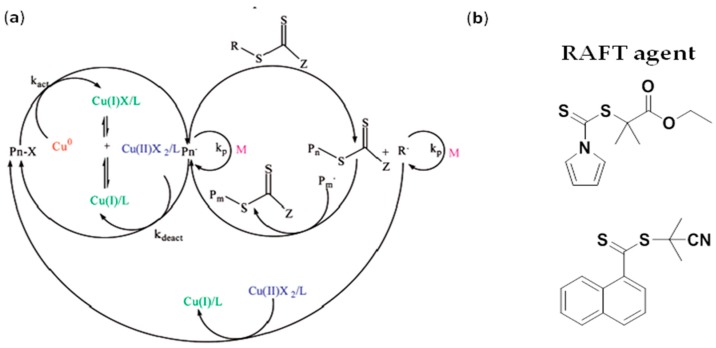
Proposed mechanism (**a**) and RAFT agents (**b**) for single electron transfer (SET)-RAFT. Reprinted with permission from [77,78,79]. Copyright 2008 American Chemical Society.

A large number of researches and applications of the metal-catalyzed living controlled radical polymerization have been published [6]. Among these studies, the supplemental activator and the reducing agent atom transfer radical polymerization (SARA ATRP) and single electron transfer-controlled living radical polymerization (SET-LRP) technology are the major researches because of their different mechanism model. However, when compared to the SARA ATRP, SET-LRP often proceeded in most polar solvents, such as DMSO, alcohols, DMF, H_2_O, and binary mixtures of these solvents with trace amount of catalysts at room temperature or below [75,76]. Especially, vinyl chloride, which cannot be prepared using current ATRP catalysts, can successfully polymerize through SET-LRP under mild reaction conditions. Many characteristics of living radical polymerization, including ultrafast polymerization rate, ultrahigh molecular weight, excellent retention of chain-end, and few side reactions can be achieved in SET-LRP. On the bright side, the successful combination of SET-LRP and RAFT technology would give a significant boost in living polymerization with controlled reaction rate [77]. In the SET-RAFT process, the radical (**R^•^** or **P_n_^•^**) is generated through SET process, in which the electron will transfer from Cu(0) to initiator (R-X) or terminated chain with the halogen end group (P_n_-X). At the same time, the oxidized metal product Cu(I)X/L will disproportionate in an instant to produce Cu(0) and Cu(II)X_2_/L. The radical species (**R^•^** or **P_n_^•^**) can be polymerized with monomer to proceed the RAFT equilibrium in the presence RAFT agent or be deactivated by Cu(II)X_2_/L to obtain the dormant state (R-X or P_n_-X) (Figure 2). The SET-LRP method in SET-RAFT partly realizes the reversible termination in RAFT process. Various monomers including styrene, acrylates and methacrylates have been polymerized with good monomer conversion rate and low dispersity on basis of the SET-RAFT technology. Owing to the catalytic reaction in click chemistry with oxidized Cu(II), well-defined polymer architecture could be constructed by cooperating of SET-RAFT and click chemistry [78]. In brief, Cu(0) that is used in the SET-RAFT polymerization could be oxidized to be Cu_2_O to loss activity, while the Cu_2_O/ascorbic acid (Vc) or CuO/Vc redox pair in click reaction could in situ generate Cu(0), which is used directly for SET-RAFT polymerization [79].

**Figure 3:**
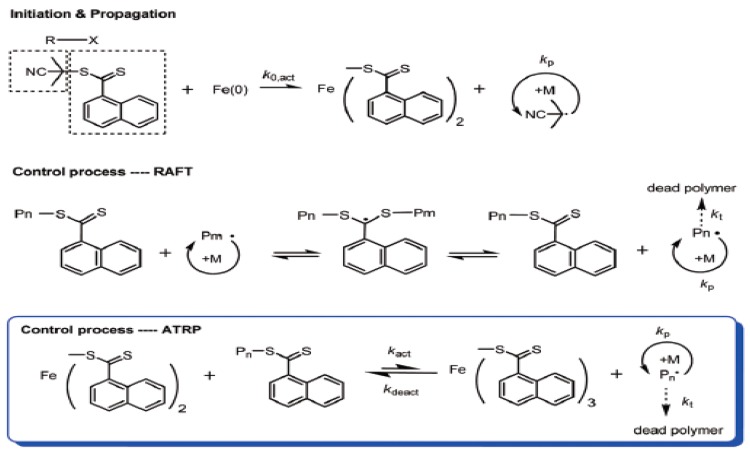
Proposed hybrid mechanism for RAFT polymerization of glycidyl methacrylate through Fe(0) catalysis initiation. Reprinted with permission from [82]. Copyright 2014 Royal Society of Chemistry.

To date, many metal-initiated RAFT systems have been published. As an example of iron (Fe), Zhu’s group reported the SET-RAFT polymerization of MMA at ambient temperature using zero-valent iron powder (Fe(0)) as a metal catalyst [80,81]. The proposed mechanism of Fe(0)-initiated RAFT is great different from that of Cu(0) catalytic system (Figure 3). The RAFT process may be in coexistence with the ATRP process, which mainly plays the control process. Using a hydrophilic RAFT agent of 4-(4-cyanopentanoic acid)dithiobenzoate (CPADB), hydrophilic monomers, including poly(ethylene glycol)monomethyl ether methacrylate (PEGMA) and DMAEMA, can go through a controllable polymerization with Fe(0) catalytic initiation. Meanwhile, Co(0), Ni(0), Zn(0) have also been introduced into SET-RAFT system for well polymerization of GMA and MMA by using 2-cyanoprop-2-yl 1-dithionaphthalate (CPDN) and cumyl dithionaphthalenoate (CDN) as RAFT agent [82].

### 3.3. Redox Initiation

When compared with thermal- and light-initiated polymerization, the redox-initiated polymerization is well developed and has been applied in a wide range of industrial products, due to its lower activation energy and higher radical generation rate under relative lower temperature. Generally, the effective redox initiator possesses a shorter induction period and higher monomer conversion. As Table 3 shows, persulfates and peroxides are two frequent oxidizing agents, which exhibit excellent redox initiation after coupling with a suitable reducing agent, such as benzoyl peroxide (BPO)/*N,N*-dimethylaniline (DMA) [83], benzoyl peroxide (BPO)/aromatic tertiary amine functionalized ATP (ATP-ATA) [84], Potassium persulfate (KPS)/L-Ascorbic acid sodium salt (NaAs) [85]. An et al. developed an aqueous redox-initiated RAFT polymerization system for fabrication of amphiphilic block copolymers [85] and highly ordered multiblock copolymer [86]. In the redox-initiated RAFT polymerization process, the permission of low reaction temperature would restrict the probability of side reaction, resulting in well living feature with high conversion (>90%) and low dispersity (<1.10) in the polymerization of NIPAM, acrylamide (AM), MMA, and styrene [87,88]. Employing ammonium persulfate (APS) and sodium formaldehyde sulfoxylate (SFS) redox initiation system, water-soluble triblock copolymer with ultra-high molecular weight (*M*n > 10^6^) could be obtained from monomers of AA and AM [89]. Meanwhile, a novel redox-initiation polymerization using Feton reaction has been reported to achieve the polymerization of DMA, NAM, and HEA, with shorter reaction time (1 min) and low polydispersity (*D* < 1.06) [90].

### 3.4. Enzyme Initiation

**Figure 4:**
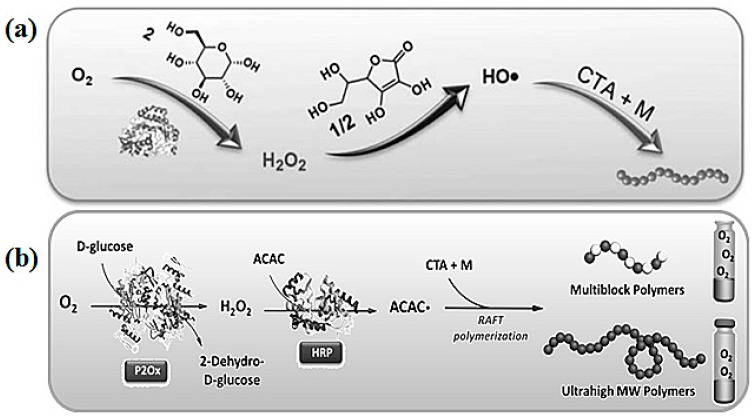
(**a**) Glucose oxidase (GOx) deoxygenation redox initiation RAFT polymerization cascade technique; (**b**) polymerization with pyranose oxidase (P2Ox)-horseradish peroxide (HRP) cascade catalysis initiation RAFT polymerization. Adapted from [99], Copyright 2016, with permission from John Wiley & Sons Inc.; and [100], Copyright 2017, with permission from John Wiley & Sons Inc.

As a green and valuable technology, the biocatalyst enzyme system has been widely utilized in the polymerization of degradable materials [91] and functional monomers [92]. Owing to the nature of high efficiency, benign, and eco-friendly, enzymatic catalysis initiation has also attracted many interests for RDRP researchers [93,94]. An et al. reported that horseradish peroxide (HRP) can initiate the RAFT polymerization of most of monomers directly under differential reaction environment [95]. In the HRP/acetylacetone (ACAC)/H_2_O_2_ ternary initiator, H_2_O_2_ catalyzed by HRP enzyme can oxidize the ACAC to produce ACAC radicals, which involves the initiation in the RAFT polymerization subsequently. In this system, advantages of fast reaction time (30 min), high monomer conversion (>90%), and low molecular weight distribution in RAFT polymerization could be observed [96]. Initially, enzyme catalysis was used to assist to degas prior to RAFT polymerization [97]. For example, the biosystem of glucose oxidase (GOx) and glucose has been employed to remove dissolved oxygen prior to PET-RAFT polymerization [74]. Recently, the smart cascade method successfully conducted redox initiation RAFT polymerization without conventional deoxygenation procedure [98]. As shown in Figure 4a, the level of dissolved oxygen can be consumed to limited level through GOx catalyzed deoxygenation reaction to produce H_2_O_2_, which can directly initiate DMA polymerization with ascorbic acid as a reductant [99]. Recently, multiblock polymers (up to 10 block) with ultrahigh molecular weight (2.3 × 10^6^ g/mol) were synthesized by using enzymatic-initiated RAFT polymerization with pyranose oxidase (P2Ox)-HRP cascade, where P2Ox primarily deoxygenates to ensure the catalysis of HRP for the generation of radical and the initiation of polymerization (Figure 4b) [100].

### 3.5. Acid Initiation

**Figure 5:**
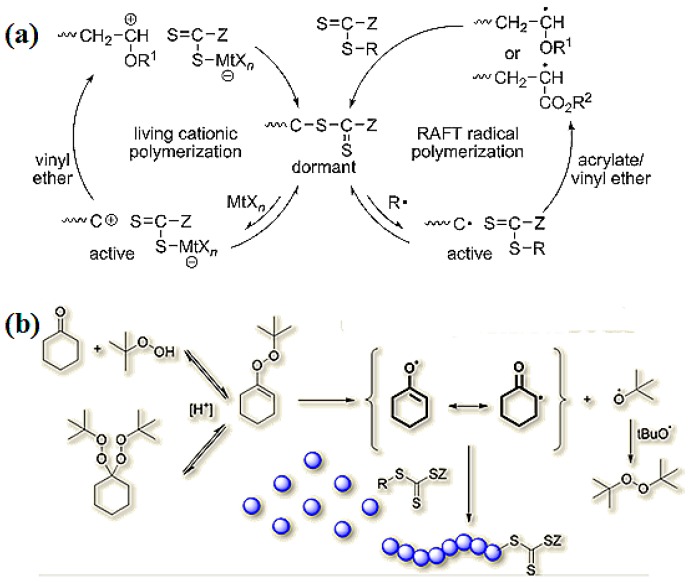
(**a**) Reversibly switching mechanism between cationic and radical polymerization of vinyl ether and acrylate monomers with the proper regulation of radical species (RC) and Lewis acids (MtXn); (**b**) Mechanism of RAFT polymerization with acid-induced cyclohexanone/tert-butylhydroperoxide initiation system at room temperature. Adapted from [103], Copyright 2014, with permission from John Wiley & Sons Inc.; and [105], Copyright 2016, with permission from American Chemical Society.

According to earlier reports, the vinyl ester is easier to be polymerized via cationic mechanism rather than radical mechanism, hence immense effort is required to obtain the copolymer containing vinyl ether by using RAFT radical approach. Lewis acids have be employed to adjust the interconversion between the RAFT radical polymerization and living cationic polymerization [101]. With this in mind, MA was homopolymerized through RAFT radical polymerization and gave a dormant species of C-SC(S)-Z. The addition of Lewis acid would activate this dormant species into cationic species for further dominating the living cationic polymerization of vinyl ester. Further research showed that the organic acids also could be used as initiator for the cationic RAFT polymerization. As an example of isobutyl vinyl ether (IBVE), it could be conducted through cationic RAFT polymerization using a handful of triflic acid as the initiator [102]. Trithiocarbonate and dithiocarbamate with electro-donating nitrogen exhibit good stabilization of cationic intermediate, which could control the molecular weights of IBVE homopolymer (PDI < 1.1) effectively in the triflylimide-initiated RAFT system. Owing to its excellent compatibility, block polymers with IBVE and MA comonomer could be synthesized through the transformation between cationic RAFT polymerization and radical RAFT polymerization (Figure 5a) [103]. Recently, the reaction between ketone and hydroperoxide participated in the organic reaction can generate the radical under acid catalysis [104]. Junkers et al. reported the utilization of p-toluenesulfonic acid to catalyze cyclohexanone/tert-butylhydroperoxide for generation ketone radical, which could be initiated RAFT polymerization of NIPAAM, styrene, and butyl acrylate (BA) with well living control, high end-group fidelities, and low dispersity [105]. However, production of ketone radical from tert-butylhydroperoxide will form the di-tert-butylperoxide, leading to the end of propagating chain in the polymerization (Figure 5b).

### 3.6. Other Initiation

Distinguish from the SET-RAFT polymerization, the metal-free dissociative electron transfer RAFT (DET-RAFT) method has been developed for the polymerization of styrene and MA at low temperature (30 °C) [106]. As a replacement of Cu(0), inorganic sulfites will transfer one electron to the RAFT agent to form radical anions through the DET process on accounts of a higher reduction potential of sulfites than that of relevant RAFT agent. This radical anion will split into a radical and an anion, following by initiation of RAFT polymerization. The polymers with narrow molecular weights distribution (PDI ≤ 1.3) and high end-group fidelity can be achieved in the DET-RAFT polymerization. Ultrasonic irradiation was once employed to initiate RAFT polymerization to graft polymers on the silica gel [107]. Inspired by the initiation system of Feton reaction catalysis [90], subsequent mechanism research illuminated that the hydroxyl radicals from water play an essential role in polymerization process. Given optimized ultrasonic parameter (frequency: 414 Hz; applied power: 40 W), the polymerization of water-soluble 2-hydroxyethylacrylate showed a high monomer conversion (>90%) within 60 min in water [108]. Besides, an excellent electrochemical-induced RAFT polymerization was reported recently [109]. The irreversible redox process of common RAFT agent makes it inapplicable for direct electrochemical activation. Using BPO or 4-bromobenzenediazonium tetrafluoroborate (BrPhN_2_^+^) as the radical initiator, the aryl radicals could be generated by electrochemical reduction for RAFT polymerization. Here, BrPhN_2_^+^ is more suitable for electrochemical-initiator than BPO, due to the similar reduction potential between BPO and workable RAFT agent. In the electrochemical-initiated RAFT polymerization, current or potential regulates the generation rate of radical as well as the polymerization degree. Until now, the homopolymers and block copolymers of BA and tBA could be fabricated with narrow molecular weight distribution successfully by electrochemical-initiated RAFT polymerization. All of the detailed summary is listed in Table 4.

### 3.7. Sulfur-Free RAFT Polymerization

**Figure 6:**
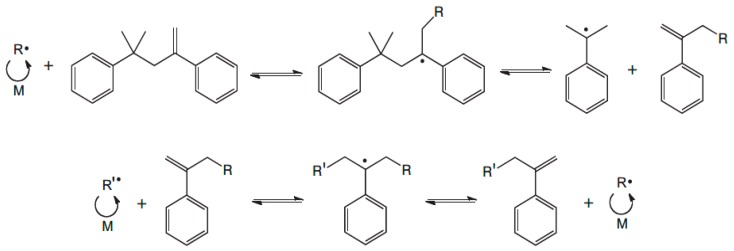
Proposed RAFT mechanism using α-methylstyrene dimer (AMSD) as RAFT agent. Reprinted with permission from [112]. Copyright 2014 Elsevier.

Aside from the research on initiation, there are some fancy findings in RAFT agent. Originally, the products from RAFT polymerization often exist in the thiocarbonate group at the ω-end, which would be destabilized with inappropriate odour and color, limiting their wide applications in many fields. The major improvements have been done by removing or functionalizing the thiocarbonate end group of RAFT products [110]. Out of these efforts, some attempts on screening of sulfur-free RAFT agents have been reported for living radical polymerization successfully [111]. When using α-methylstyrene dimer (AMSD) as RAFT agent, RAFT-like process is observed in the polymerization (Figure 6) [112]. However, the tertiary radical generated from AMSD is relatively unstable, resulting in uncontrollable poly(methacrylate)s with large molecular weight distribution. Methanol also has been investigated as RAFT agent (optimal concentration of 6.5 mol %) for the synthesis of poly(4-methoxystyrene) (PDI ≤ 1.2) bearing methoxy end group [113].

**Figure 7:**
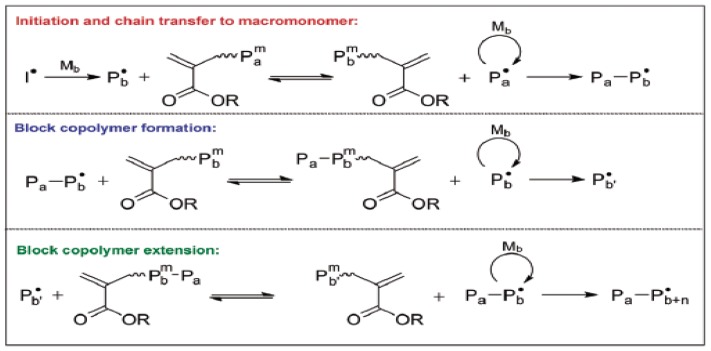
Proposed mechanism of sulfur-free RAFT process. Reprinted with permission from [115]. Copyright 2016 Royal Society of Chemistry.

Recently, Haddleton and coworkers developed the sulfur-free RAFT emulsion polymerization for the preparation of methacrylic block copolymers [114,115]. Vinyl-terminated poly(methyl methacrylate) (PMMA) oligomer could be fabricated through the method of catalytic chain transfer polymerization (CCTP) in emulsion [116]. These unsaturated oligomers presented appealing chain transfer constants, which could be continue to be used as chain transfer agent for RAFT polymerization of MMAs, following the addition of potassium persulfate as radical resource (Figure 7) [117,118]. Based on this strategy, the heneicosa and tetracosa quasi multiblock poly(butyl methacrylate) could be achieved with narrow molecular weight distribution (PDI ≤ 1.21). The copolymer of benzyl methacrylate, 2-ethyl hexyl methacrylate, and butyl methacrylate could also be prepared with extremely high conversions (>99%). With the increasing of polymerization degree (DP_n_ = 45) in each block, higher molecular weight hepta-block copolymers were synthesized. Furthermore, the product at a large scale (up to 80 g in a 0.5 L double jacket reactor) could be gained with a low polydispersity (PDI ≤ 1.24), as well as high molecular weight (*M*n = 41,300 g/mol). These attractive results suggest the sulfur-free RAFT technology for wide prospects in application.

## 4. Application

Since conducting polyacetylene was firstly reported by Heeger and coworkers in 1977 [119], optoelectronic polymers, as a new type of organic functional materials, have great research significance and application potential because of their excellent optoelectronic properties, easily preparation, and low cost. However, great efforts have been solely devoted to the synthesis of functional polymers or the optoelectronic applications of existing materials, there have been only few other interdisciplinary efforts in the field of optoelectronics. D’hooge et al. have attempted to carry on interdisciplinary to combine the simulation and radical polymerization [120,121,122], which greatly empowers us to understand and control radical polymerization. According to the experiment data, the kinetic models has been established to evaluate two different precursor routes for the synthesis of poly(*p*-phenylene vinylene) (PPV) derivative that is often as active material for the optoelectronic devices, and the simulation results contribute to our selection of optimal reactants and reaction conditions [123,124].

However, RAFT polymerization with many good merits also satisfies the interdisciplinary research with an optoelectronic field. First, the undesired metal catalyst can be avoided to use for the synthesis of functional polymers. At the same time, narrow molecular weight distribution will greatly reduced the low molecular weight “impurities” which can degrade performance of semiconductors as electron or hole traps. Then, simplified preparation methods of multiblock copolymers and complex polymer architectures allow us to design and prepare multi-functional macromolecular materials. Finally, RAFT agent can also play the role of binder between organic materials and inorganic/organic materials. All of this suggests that it’s greater prospects for the applying RAFT polymerization to the preparation of optoelectronic materials. The use of functional polymer from RAFT polymerization in the electro-optic field has been prospering in recent years and plenty of related researches have been reported [125,126].

**Figure 8:**
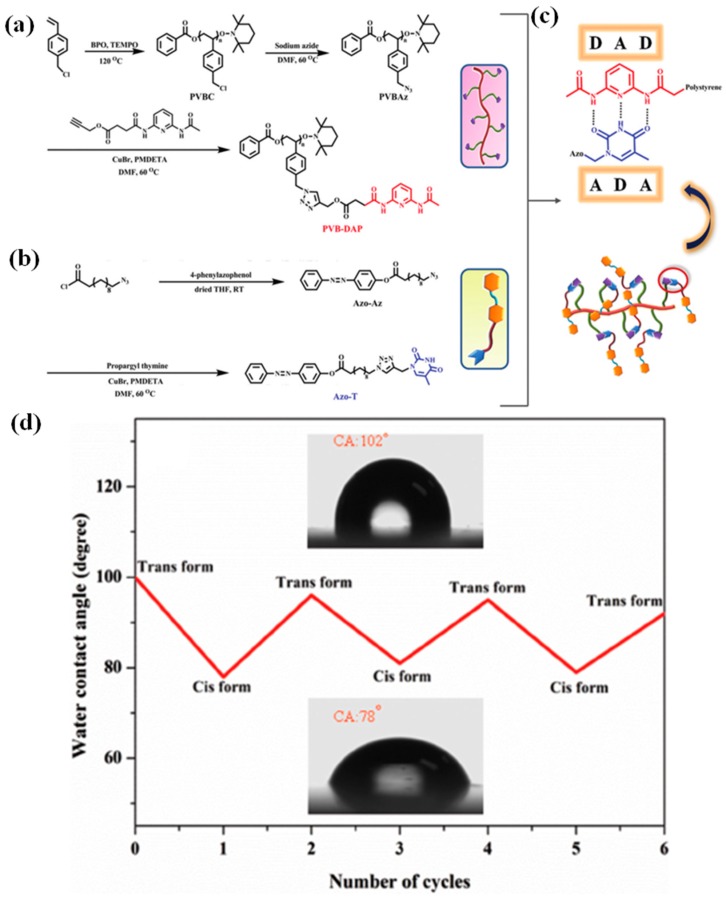
Synthesis of (**a**) PVB-DAP; (**b**) Azo-T; and (**c**) hydrogen-bond based supramolecular complex; (**d**) The variations of water contact angles of PVB-DAP/Azo-T hydrogen-bond based supramolecular complexes after several cycles between UV irradiation and being placed in the dark. Reprinted with permission from [127]. Copyright 2015 Royal Society of Chemistry.

**Figure 9:**
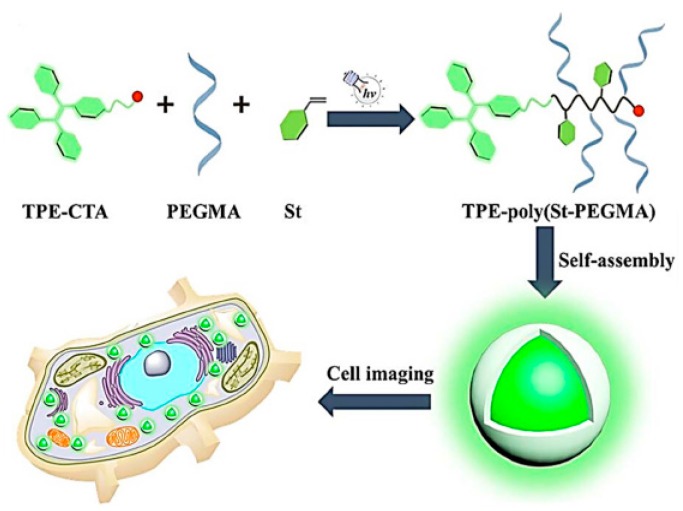
The preparation of TPE-poly(St-PEGMA) fluorescent polymeric nanoparticles (FPNs) and their cell imaging applications. Reprinted with permission from [130]. Copyright 2018 Elsevier.

**Figure 10:**
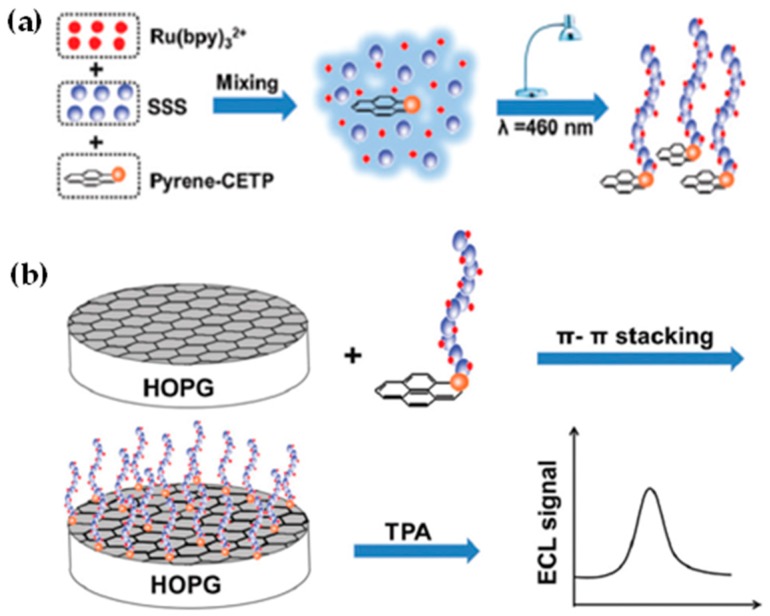
(**a**) The fabrication of pyrene-terminated PSS via PET-RAFT polymerization and production of Ru(bpy)_3_^2+^ adsorbed PSS concurrently; (**b**) Diagrammatic sketch for the fabrication of Ru/PSS based solid-sate ECL sensor. Reprinted with permission from [133]. Copyright 2016 Royal Society of Chemistry.

Based on the trans-cis isomerization of azobenzene derivatives under light irradiation, Kuo et al. reported the synthesis of functional material, named PVB-DAP, by RAFT polymerization, which could further interact with thymine-functionalized azobenzene to give a supramolecular complex. This supramolecular material featured the “recordable” and “rewritable” photo-responsive properties, which implied its promising application in optical devices (Figure 8) [127]. The Silicon nanocrystals as the photoluminescent material often suffer from oxidation-induced poor emission performance. Rieger et al. demonstrated the surface modification of silicon nanocrystals by using RAFT polymerization of polystyrene to form core-shell hybrid product, which provided the more stable photoluminescence properties, due to the protection of polymer shell against oxidation [128]. The amino-containing aggregation-induced emission (AIE) fluorescent molecule could be introduced into the side chain of hydrophilic polymers (PEGMA-*co*-UCL) via the combination of RAFT polymerization and mercaptoacetic acid locking imine (MALI) reaction in one pot, the resulting luminescent polymer with AIE characteristic exhibited the ideal candidates for cell imaging based on its intensive emission and excellent biocompatibity [129]. Meanwhile, AIE also could be introduced to couple with RAFT agent, which then was used to synthesize the AIE active fluorescent polymeric nanoparticles. Zhang et al. found that the AIE-actived polymers with enhanced fluorescence intensity and photostability are beneficial for the application in biological imaging (Figure 9) [130]. Similarly, an amphiphilic copolymer has been synthesized from hydrophobic fluorescein methacryloyl monomer and hydrophilic poly(ethylene glycol) monomethacrylate. The fluorescent organic nanoparticles that were obtained from the self-assemble of this copolymer showed the good biocompatibility and excellent florescence, rendering it a promising material for the application in cell imaging [131]. Kong et al. synthesized an amphiphilic poly(*N*-vinyl carbazole) (PVK)-based block copolymer with the pendent of tris(8-hydroxyquinoline) aluminum (Alq_3_) using trithiocarbonate terminated PEG as a RAFT agent [132]. The resonance energy transfer from PVK to Alq_3_ could give raise to the enhancement of emission from Alq_3_, which made it an excellent material as electron-transporting and emission layer in OLED. Ru(bpy)_3_^2+^ not only is an efficient catalyst for PET-RAFT polymerization, but also can be used as a electrogenerated chemiluminescent (ECL) reagent. Liu et al. presented the preparation of pyrene-modified poly(sodium *p*-styrene sulfonate) (PSS) through PET-RAFT approach using Ru(bpy)_3_^2+^ as catalyst, which could be directly used to fabricate the solid-state ECL sensor through the electrostatic interaction between Ru(bpy)_3_^2+^ and PSS. This ECL sensor exhibited the high sensitivity and long term stability (Figure 10) [133].

**Figure 11:**
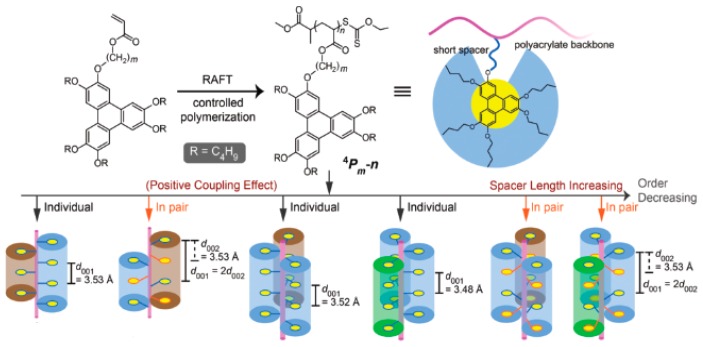
Well-defined side-chain discotic liquid crystal polymers ^4^P_m_-n synthesized by RAFT polymerization and the spacer length-dependent self-organized superstructures, including columnar plastic phases via butoxy-substituted triphenylene (TP) in pairs stacking modes and columnar liquid crystal mesophases from single TP discotic units. Reprinted with permission from [134]. Copyright 2017 Royal Society of Chemistry.

**Figure 12:**
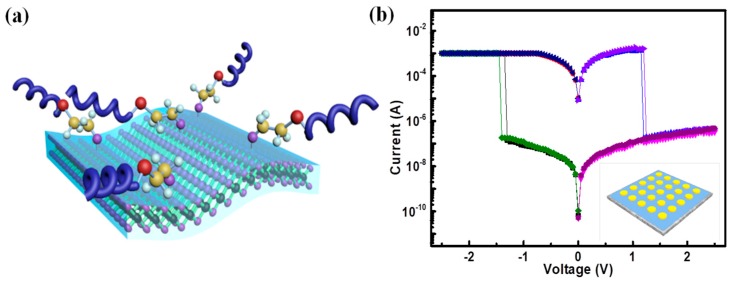
(**a**) Covalent PVK-modified MoS_2_ nanosheets and (**b**) Current-voltage (I–V) characteristics of ITO/MoS_2_-PVK/Au memory device. Reprinted with permission from [144].

For electric applications, Chen et al. reported the synthesis of butoxy-substituded triphenylene (TP)-based polymers by RAFT strategy. Owing to the existence of rigid triphenylene, homopolymer could self-assemble into various ordered columnar plastic phases with highest hole mobility of 0.1 cm^2^ V^−1^ s^−1^ (Figure 11) [134]. Subsequently, they developed a cross-linked copolymer with high dielectric constant, which has been demonstrated the superior low-voltage operation performance in the application of organic field-effect transistors [135]. The core-shell nanoparticles can also give the excellent dielectric performance. Jiang and coworkers synthesized a series of core-shell polystyrene (PS)@BaTiO_3_ nanocomposites by grafting PS from the BaTiO_3_ surface through in situ RAFT polymerization [136]. The different thickness of PS shell could regulate the dielectric constant of nanoparticles, and the dielectric constant of core-shell BaTiO_3_@PS is 24, which is roughly eight times larger than that of separate PS (2.76). Meanwhile, the extremely low dielectric loss of BaTiO_3_@PS had been kept. Chen et al. reported the preparation of cross-linked core-shell block copolymer, poly[poly(ethylene glycol)methylether methacrylate]-block-poly(2,5-dibromo-3-vinylthiophene) (poly(PEGMA)*_m_*-*b*-poly(DB3VT)*_n_*) on the basis of RAFT technology, which was applied firstly as charge-storage materials for non-volatile OFET memory devices [137]. They demonstrated the size of the cross-linked nanocomposites and the molecular weights of poly(PEGMA) segments have positive correlation, while the mean diameter of poly(DB3VT) segments can effectively adjust the optical and electronic memory properties. The maximum hole-trapping capacity of the OFET memory device could be achieved by using the block copolymer of poly(PEGMA)*_m_*-*b*-poly(DB3VT)*_n_* with a monomer molar ratio of 77:23 (*m*:*n*). Recently, Chen’s group reported two kinds of RAFT strategy for the preparation of poly(*N*-vinylcarbazole) (PVK)-modified graphene oxides (GO). One was “Graft to” method, in which PVK was prepared by traditional RAFT approach by using S-1-dodecyl-S’-(α,α’-dimethyl-α-aceticacid) trithiocarbonate (DDAT) as a RAFT agent, and the product of DDAT-PVK can directly bond onto the isocyanate-functionalized GO with covalent amido linkage [138]. Another was “Graft from” method, where covalent DDAT-functionalized GO (DDAT-GO) was firstly fabricated and used as macroRAFT agent for the polymerization of *N*-vinylcarbazole through RAFT technology [139]. Both two methods could improve the solubility of GO, which realized the solution process of GO-based materials. When PVK-GO was used for the polymer memories, the device presented an excellent bistable electrical switching and nonvolatile memory effect. Emrick et al. elaborated the preparation of tetrathiafulvalene (TTF)-based polymers using RAFT polymerization, which was then used for the non-covalent surface functionalization of MoS_2_ to decrease its work function and modulate the electronic band structure [140]. In the same year, Chen and coworkers firstly synthesized covalent PVK-modified MoS_2_ nanosheets through RAFT polymerization, which exhibited an improvement of solubility in organic solvent. As electric devices, the enhancement of charge transfer of MoS_2_-PVK guaranteed the excellent optical limiting response and nonvolatile rewritable memory performance (Figure 12) [141,142]. Similar results have also been found in covalent polyacrylonitrile (PAN)-functioned MoS_2_ nanosheets (MoS_2_-PAN) [143,144]. These findings give the growing evidences of RAFT polymerization for huge potential in the optoelectronic field.

## 5. Conclusions

From the basic discoveries to practical applications, RAFT polymerization has become one of the indispensable parts in polymer chemistry. Large number of theoretical and empirical researches are relevant to RAFT polymerization during the past 20 years. New RAFT techniques have been proposed to make up the deficiency of conventional RAFT methods, such as different initiation system (e.g., photo, metal, enzyme, redox, and acid) and sulfur-free RAFT polymerization. Although a great deal of progress has already been achieved, several major challenges still exist for these novel RAFT techniques to achieve their broad applications in optoelectronic field, including new RAFT polymerization system, convenient operating condition without anaerobic treatment, introduction of various optoelectronic properties onto backbone of polymer by RAFT polymerization, and investigation of complex architecture and self-assembly of polymers synthesized by RAFT polymerization on their optoelectronic performances. With the continuous advancement of RAFT technology, we believe that RAFT technology will become the most powerful tool available for design and fabrication of complex architecture polymers for optoelectronic applications.

## Figures and Tables

**Table 1:** RAFT agents and monomers for direct photodissociation RAFT.

Photoiniferter	Polymerization
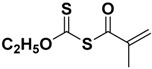	Methyl methacrylate (MMA); Methyl acrylate (MA); Styrene [47]
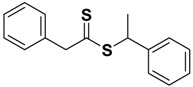	Styrene [48]
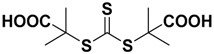	Acrylic acid (AA) [50]
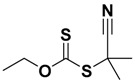	Vinyl acetate (VAc) [52]
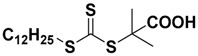	MA [54]
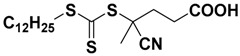	Butyl methacrylate (BMA) [55,57]; MMA [57]; MA [57]
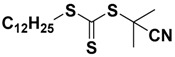	MA [57]
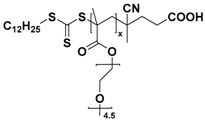	Benzyl methacrylate (BzMA) [51]

**Table 2:** RAFT agents, photoredox catalysts and monomers for PET-RAFT.

RAFT agent	Photoredox Catalyst	Monomer
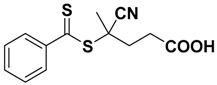	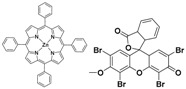 ZnTPP [63]; Eosin Y [66] 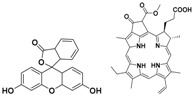 Fluorescein [66]; Pheo A [70]; fac-[Ir(ppy)_3_] [58]; Ru(bpy)_3_Cl_2_ [61]; TiO_2_ [68]; Bacteriochlorophyll a [71]	MMA [58,63,66,68,71]; *N*-(2-hydroxypropyl) methacrylamide (HPMA) [58]; Oligo(ethylene glycol) methyl ether acrylate (OEGA) [61]; Oligo(ethyleneglycol) methyl ether methacrylate (OEGMA) [61]; Hydroxylethyl methacrylate (HEMA) [66]; *N*,*N*-dimethyl aminoethyl methactylate (DMAEMA) [66]; Pentafluorophenyl methacrylate (PFDMA) [66]; Methacrylic acid (MAA) [66]; Hydroxylpropyl methacrylamide (HPMA) [66]; Glycidyl methacrylate (GMA) [66]; Oligo-ethylene glycol methyl ether methacrylate (OEGMA) [66]; *O*-nitrobenzyl methacrylate(NBMA) [70]
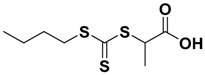	fac-[Ir(ppy)_3_] [58]; Ru(bpy)_3_Cl_2_ [61]; 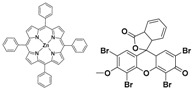 ZnTPP [63]; Eosin Y [67]	MA [58,63]; Styrene [58,63]; Oligo(ethyleneglycol) methyl ether acrylate (OEGA) [58,61]; Tert-butyl acrylate (tBA) [58]; *N*-isopropylacrylamide (NIPAAM) [58,61,63]; Isoprene [58]; *N*,*N*-dimethyl acrylamide (DMA) [58,61,63]; *N*,*N*’-diethylacrylamide (DEA) [61]; 2-(dimethylamino)ethyl acrylate (DMAEA) [63]; Polyethylene glycol (PEG)-based acrylamides [67]
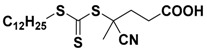	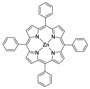 ZnTPP [63,70,72]	MMA [63]; *N*-(2-hydroxylpropyl) methacrylamide (HPMA) [63]; *O*-nitrobenzyl methacrylate (NBMA) [70]; 2-(methylthio) ethyl methacrylate; (MTEMA) [72]
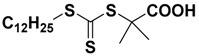	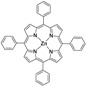 ZnTPP [63]	MMA [63]
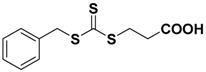	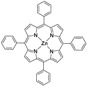 ZnTPP [63]; fac-[Ir(ppy)_3_] [58]	Styrene [63]; MA [58]
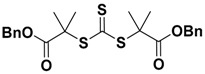	10-phenylphenothiazine (PTH) [59]	NIPAAM [59]; DMA [59]; tBA [59]; Ethylene glycol methyl ether acrylate (EGMEA) [59]
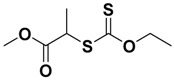	fac-[Ir(ppy)_3_] [58]	VAc [58]; Vinyl pivalate (VP) [58]; Dimethyl vinylphosphonate (DVP) [58]; *N*-vinyl pyrolidinone (NVP) [58]
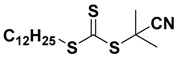	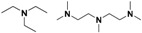 TEA [65]; PMDETA [65]; 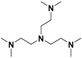 *g*-C_3_N_4_ [73]; Me_6_TREN [65]	MMA [65]; BuMA [65]; Ethyl methacrylate (EMA) [65]; MA [73]; DMA [73]
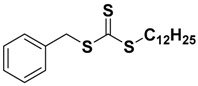	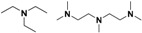 TEA [65]; PMDETA [65] 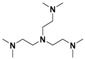 *g*-C_3_N_4_ [73]; Me_6_TREN [65]	MA [65]; Styrene [65]; NIPAAM [65]; MA [73]; DMA [73]
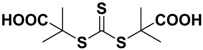	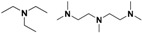 TEA [65]; PMDETA [65]; 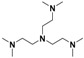 Me_6_TREN [65]	OEGMA [65]
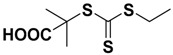	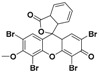 Eosin Y [69]	DMA [69]; 2-hydroxyethyl acrylate (HEA) [69]; Acid functional 2-acrylamido-2-methyl-1-propane-sulfonic acid (AMPS) [69] NIPAAM [69]; Sodium styrenesulfonate (NaSS) [69]; Poly(ethylene glycol) acrylate (PEGA) [69]
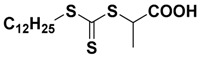	*g*-C_3_N_4_ [73]	MA [73]; DMA [73]
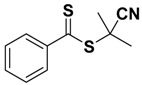	*g*-C_3_N_4_ [73]	MMA [73]
R: 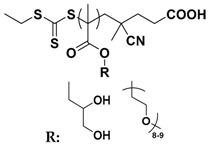	Sodium phenyl-2,4,6-trimethyl-benzoylphosphinate (SPTP) [74]	HPMA [74]

**Table 3:** RAFT agents, redox initiator and monomers for RAFT via redox initiation.

RAFT Agent	Redox Initiator	Monomer
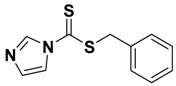	BPO/DMA [83,88]	4-azidophenyl methacrylate (APM) [83]; MA [83,88]; MMA [83,88]; Styrene [83,88]
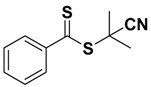	BPO/DMA [83,88]; BPO/ATP-ATA [84]	4-azidophenyl methacrylate (APM) [83]; MA [83,88]; MMA [83,84,88]; Styrene [83,88]
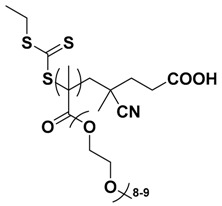	KPS/NaAs [85]	2-methoxyethyl acrylate (MEA) [85]; Poly(ethylene glycol) methyl ether methacrylate (PEGDA) [85]
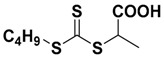	Tert-butyl hydroperoxide (TBHP)/ascorbic acid (AsAc) [86]; TBHP/Bruggolite^®^ FF7 (Brug 7) [86]	4-acryloylmorpholine; HEA; DEA; EGMEA; AA; MA; NIPAAM; PEGA; DMA; 2-carboxyethylacrylate (CEA) [86]
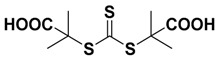	K_2_S_2_O_8_/Na_2_S_2_O_3_ [87]; H_2_O_2_/Fe^2+^ [90]	NIPAAM [87]; AM [87]; DMA [90]; NAM [90]; HEA [90]
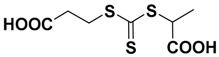	APS/SFS [89]	AA [89]; AM [89]

**Table 4:** RAFT agents, initiator and monomers for RAFT polymerization.

RAFT Agent	Initiator	Monomer
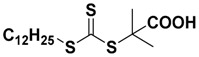	Na_2_S_2_O_4_ [106]; ultrasonic irradiation [107]; electronreduction of BPO or BrPhN_2_^+^ [109]	Styrene [106]; MA [106,107,109]; MMA [109]; BA [109]
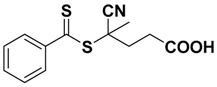	Na_2_S_2_O_4_ [106]; electronreduction of BPO or BrPhN_2_^+^ [109]	MMA [106]; GMA [106]; 2-(diisopropylamino) ethyl methacrylate (DPA) [106]; DMAEMA [106]; MMA [109]; BA [109]
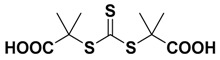	ultrasonic irradiation [108]	HEA; OEGA; DMA; *N*-acryloylmorpholine (NAM); OEGMA [108]
